# Racial and ethnic disparities in the management of risk factors in individuals with atrial fibrillation: A narrative review

**DOI:** 10.1016/j.ajpc.2025.101361

**Published:** 2025-11-28

**Authors:** Anthony D’Oro, Zoee D’Costa, Jasmyn J. Tang, Stephen Min, Larry R. Jackson, Utibe R. Essien

**Affiliations:** a University of California, Los Angeles, Department of Medicine, 757 Westwood Plaza, Los Angeles, CA 90095, USA; b University of California, Los Angeles, Division of General Internal Medicine and Health Services Research, 1100 Glendon Ave, Suite 850, Los Angeles, CA 90024, USA; c Duke Clinical Research Institute, 300 W Morgan St, Durham, NC 27701, USA; d Center for the Study of Healthcare Innovation, Implementation, and Policy at the Greater Los Angeles Veterans Affairs, 11301 Wilshire Blvd, Los Angeles, CA 90049, USA

**Keywords:** Atrial fibrillation, Obesity, Hypertension, Diabetes, Sleep apnea, Alcohol, Smoking, Disparities, Pharmacoequity

## Abstract

Atrial fibrillation (AF) is the most prevalent arrhythmia, affecting approximately 10 million individuals in the United States and nearly 60 million people globally. Significant racial and ethnic disparities exist in rates of complications for individuals with AF. Compared to White patients, Black and Hispanic patients are significantly more likely to experience stroke and death. While research has examined pharmacoequity in receipt of stroke-preventing therapies among individuals with AF, limited literature exists examining disparities in risk factor management for AF complications. The American College of Cardiology / American Heart Association Joint Committee on Clinical Practice Guidelines have identified ten risk factors whose effective management reduces the recurrence of AF, symptom burden, and complications. Six cardiometabolic risk factors, selected given the availability of evidence-based therapies to manage them, will be discussed herein including obesity, hypertension, diabetes, obstructive sleep apnea, alcohol consumption, and tobacco use. We seek to highlight the data examining how risk factors contribute to poor outcomes in patients with AF and to summarize the data on racial and ethnic disparities in the management of those risk factors in this patient population. Lastly, we provide a call to action to achieve pharmacoequity in AF risk factor management and improve downstream outcomes.

**Figure:**
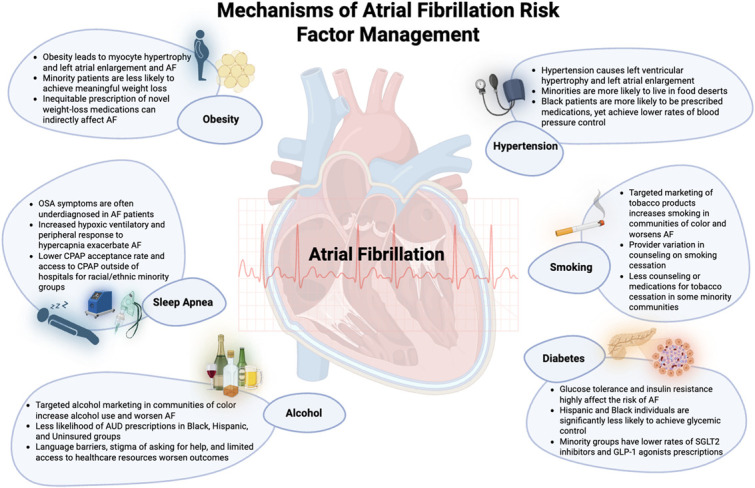
Central Illustration.

## 1 Introduction

Atrial fibrillation (AF) is the most prevalent arrhythmia, affecting approximately 10 million individuals in the United States [1] and nearly 60 million people globally [2]. According to estimates from the Framingham Heart Study, age-adjusted prevalence of AF increased from 20.4 to 96.2 cases per 1000 person years between 1958 and 2007 [3]. Moreover, the prevalence of AF is expected to double in the next 25 years [4]. Complications from AF include heart failure, ischemic heart disease, and stroke, and lead to approximately 460,000 hospitalizations, 80,000 deaths [5], and an estimated healthcare cost of $26 billion each year [6].

Whereas prior work has been mixed in reporting variation in incidence of AF by race and ethnicity [7,8], significant disparities exist in rates of adverse outcomes for individuals with AF. Compared to non-Hispanic White (White) patients, non-Hispanic Black (Black) and Hispanic patients with AF are significantly more likely to experience stroke and death[[9], [10], [11]]. Compared to White patients, Black patients with AF experience approximately double the rates of hemorrhagic or ischemic stroke [12]. Black patients with AF also suffer higher rates of heart failure, coronary artery disease, and death [12,13]. Higher rates of stroke have also been observed among Black Medicare beneficiaries [10] and Black enrollees in the Veterans Health Administration (VA) with diagnosed AF [14].

Despite the growing literature describing outcome differences among individuals with AF by race and ethnicity, the determinants of these disparities remain underexplored. Prior work has primarily examined differences in anticoagulation as a driver of disparities, with multiple analyses demonstrating lower receipt of direct oral anticoagulant (DOAC) therapy in particular among minoritized groups[[15], [16], [17]]. Among those who are prescribed oral anticoagulation therapy, Black and Hispanic patients are more likely to be offered warfarin vs. DOACs [16,18,19]. Beyond anticoagulation, minoritized patients are less likely to be offered anti-arrhythmic medications or advanced therapies like catheter ablation aimed at restoring normal sinus rhythm[[20], [21], [22]]. A nationwide U.S. analysis of patients with AF found Black race and Hispanic ethnicity be to significantly associated with 11 and 27 % lower odds of catheter ablation receipt respectively [22]. These disparities are critical as prior work has shown significant improvement in AF-associated outcomes (e.g., stroke, bleeding, cardiac arrest) and mortality in minoritized patients receiving catheter ablation compared with non-minoritized groups [23].

Representation of racial and ethnic minoritized groups in clinical trials of AF management has historically been low. This poor representation is often the result of historical exploitation and healthcare discrimination and, despite greater societal awareness, has not significantly improved over time [24]. In the landmark trials of DOAC medications, Black patients represented only 2 % of the study population while Hispanic patients represented only 3.9 %, far lower than their national representation[[25], [26], [27]]. In the CABANA (Catheter Ablation Versus Antiarrhythmic Drug Therapy for Atrial Fibrillation) trial comparing catheter ablation to anti-arrhythmic medications, Black patients represented only 3.5 % of the study population and minoritized groups in total represented <10 % of the study population [28]. A single-center study of patients who received ablation therapy from 2000–2009 included a patient population that was 93 % White compared to 2 % Black and fewer than 1 % Hispanic or Asian patients each [29]. These data suggest a critical opportunity to advance AF care in diverse populations.

Robust literature exists to support the association between AF recurrence and cardiometabolic risk factors including data from the diverse Cohorts for Heart and Aging Research in Genomic Epidemiology (CHARGE-AF) [30]. While the initial detection of AF may be hindered by its paroxysmal nature, varied disease burden, and varied symptom manifestation, all patients with AF should be screened for risk factors and comorbidities whose effective management can greatly alter the disease course and mitigate future complications [31]. The American College of Cariology / American Heart Association Joint Committee on Clinical Practice Guidelines has identified ten risk factors or management strategies whose effective management and utilization has demonstrably reduced recurrence of AF, symptom burden, or reduced complications [31]. Six of the ten cardiometabolic risk factors will be discussed including: obesity, hypertension, diabetes mellitus, obstructive sleep apnea, alcohol consumption, and tobacco use (**Central Illustration)**). We selected these six specific risk factors because of their strong association with daily clinical practice and the evidence-based therapies that exist to manage them, thereby providing clinicians with tangible opportunities to reduce complications of AF.

In this Review, we seek to highlight mechanisms by which risk factors contribute to AF incidence and adverse outcomes in patients with AF. Additionally, we seek to summarize the data on racial and ethnic disparities in the incidence and management of risk factors in patients with AF, including an emphasis on how minoritized groups have been underrepresented in AF clinical trials. We also provide a call to action for increased investigation on the determinants of disparities in the incidence and management of risk factors associated with AF. Such research is needed to guide future, targeted, individual- and population-level interventions and advance quality and pharmacoequity of care for AF.

## 2 AF and obesity

Obesity is a cardiometabolic syndrome that has steadily grown in prevalence in the U.S. and globally. Since 1970, the prevalence of Americans with a body mass index (BMI) greater than 25 has grown from ∼15 % to 42 % with varying rates by race and ethnicity [32,33]. In a similar period, the prevalence of AF has nearly quadrupled.

Several pathophysiologic pathways link obesity with AF (Fig. 2). The increased circulating volume associated with obesity places more demand on the left atrium and left ventricle, leading to myocyte hypertrophy and left atrial enlargement [34]. Patients with AF have been shown to have increased myocardial fibrosis and differential fatty deposition in the myocardium, potentially acting as a substrate for arrhythmia [35]. Epicardial adipose tissue has been shown to have a step-wise relationship with the risk of AF [36,37].

Research examining the relationship between obesity and adverse outcomes in AF have been mixed, suggesting an “obesity paradox.” [38] A post hoc analysis of the Atrial Fibrillation Follow-up Investigation of Rhythm Management (AFFIRM) study found that patients who were overweight (hazard ratio [HR] 0.64; 95 % confidence interval [CI], 0.48–0.84; *P*=.001) and obese (HR 0.80; 95 % CI, 0.68–0.93; *P*=.005) had lower all-cause mortality as compared to those with normal weight [39]. The ENGAGE AF‐TIMI 48 (Effective Anticoagulation With Factor Xa Next Generation in Atrial Fibrillation–Thrombolysis in Myocardial Infarction 48) trial showed that overweight and obese patients with AF had lower risk of stroke and systemic embolism compared with normal weight patients [40]. In contrast, a Danish analysis found significantly higher rates of ischemic stroke, thromboembolism, and death in overweight and obese individuals compared to those with normal weight [41].

Despite the apparent obesity paradox, reducing BMI in patients who are obese with AF has been shown to reduce AF recurrence and symptom-burden including in the LEGACY (Long-Term Effect of Goal directed weight management on Atrial Fibrillation Cohort) and SORT-AF (Supervised Obesity Reduction Trial for AF Ablation Patients) studies [42,43]. A study of 150 patients in Australia demonstrated that an active weight-loss intervention and sustained weight loss resulted in reduced self-assessed symptom burden, less time in AF based on electrocardiographic monitoring, reduced left atrial size, and reduced interventricular thickness [44]. Another study demonstrated a step-wise, direct relationship between weight and AF severity, with greater weight loss able to reverse classification of AF from persistent to paroxysmal or no AF with greater success than moderate or no weight loss [45].

Targeted weight loss programs are efficacious in reducing BMI and improving overall health[[46], [47], [48]]. However, this research suggests that minoritized populations may not gain the same degree of weight loss compared to White patients [48]. The Prediabetes Informed Decision and Education (PRIDE) trial showed that Hispanic patients were less likely to achieve weight loss after participation in a behavioral intervention program, citing barriers including inconvenient location of classes, limited time to attend classes, and affordability of adjunct interventions like gym memberships [49,50]. Another study showed that Black women participating in weight loss intervention groups were less likely to achieve the same amount of weight loss as women from different racial and ethnic groups [51].

For individuals in whom behavioral intervention is insufficient at achieving weight loss, pharmacologic therapy is recommended [32,52]. Historical medications to achieve weight loss include orlistat, phentermine, and bupropion, with efficacy rates ranging from 3–8 % of total body weight loss (TBWL); while newer incretin-based therapies like glucagon-like peptide-1 receptor agonist (GLP-1RA) medications have demonstrated efficacy ranging from 10–18 % TBWL [53,54]`.

Nonetheless, there are inequities in receipt of novel therapeutics for obesity [16,55]. A 2021 study showed that Black, Hispanic, Asian, and lower-income patients were less likely to be prescribed GLP-1RA medications than White or higher income patients [56]. Another analysis showed 30 % fewer Black individuals were prescribed GLP-1RA therapies from 2013–2019 than White individuals [57]. These findings were corroborated in a VA analysis of over 1.9 million patients which showed that Black and American Indian patients were less likely to be prescribed GLP-1RA therapies than their White counterparts [58]. Moreover, in landmark trials of GLP-1RA therapies evaluating cardiovascular outcomes, racial and ethnic minoritized groups were largely underrepresented [59,60].

The inequitable prescription of these novel weight-loss medications has been directly linked to primary incidence of AF. A 2024 meta-analysis of 10 randomized control trials demonstrated that prescription of GLP-1RA therapies is associated with 42 % lower risk of incident AF [61]. Moreover, GLP-1RAs have been shown to reduce recurrence of AF after catheter ablation [62]. Together, these outcomes data suggest an urgent need to address obesity as a driver of adverse outcomes in AF, yet, data are scarce on how management of obesity may influence downstream AF outcomes including stroke and mortality. Furthermore, how such management may differ by race and ethnicity is to date unknown and warrants further study.

## 3 AF and hypertension

Approximately 46 % of U.S. adults carry a diagnosis of hypertension[[63], [64], [65]]. Overall, Asian individuals have a 36–45 % prevalence of hypertension among women and men, respectively, compared to 42–44 % among Hispanic, 41–47 % among White, and 56–59 % among Black individuals. Importantly, 80 % of patients with AF have comorbid hypertension, making it a prime target of risk factor management [66].

There are several mechanisms by which hypertension is thought to contribute to incidence of AF (Fig. 2). Hypertension has been demonstrated to cause left ventricular hypertrophy and left atrial enlargement due to elevated filling pressures in the left ventricle. In the Framingham Heart Study, for each 4 mm increase in thickness of the left ventricular wall, incidence of AF increased by 28 % [67]. Similarly, for each 5 mm increase in left atrial size, prevalence of AF increased by 39 % [67].

Given the high comorbid prevalence of hypertension with AF, control of hypertension is essential to minimizing risk of AF incidence and recurrence. Yet while myriad studies have demonstrated an association between hypertension and AF incidence [[68], [69], [70], [71]], few have linked hypertension directly to adverse AF outcomes. In a post-hoc analysis of the Apixaban for Reduction in Stroke and Other Thromboembolic Events in Atrial Fibrillation (ARISTOTLE) trial, researchers found that an elevated blood pressure measurement at any point during the trial, was associated with a significantly higher rate of stroke or systemic embolism (HR, 1.53; 95 % confidence interval [CI], 1.25–1.86), hemorrhagic stroke (HR 1.85; 95 % CI, 1.26–2.72), and ischemic stroke (HR, 1.50; 95 % CI, 1.18–1.90) [72]. A separate analysis of patients with AF in Korea found that ischemic stroke and systemic embolism occurred in 9.8 % of hypertensive patients, compared with 4.6 % of non-hypertensive patients, with an increased risk observed in the highest quartile of systolic blood pressures [73].

Control of blood pressure is disparate between racial and ethnic subgroups, with correlate consequences for outcomes of stroke, cardiovascular disease, and death. Black patients have been shown to be less likely to achieve blood pressure control than White patients [74]. Control of blood pressure is also worse among Asian and Hispanic patients, with rates of control between 39.9 % and 43.5 %, respectively, compared to 59.1 % for White adults [75]. Hypertension control is often multimodal, including lifestyle interventions such as weight loss, limitation of alcohol consumption, participation in physical activity, and dietary modification [63]. Racial variation in uptake of lifestyle interventions may account for the disparities observed in optimal blood pressure control [76]. The PRIDE Trial demonstrated that Black and Hispanic patients face social barriers to participation in weight loss programs, including cost, geographic availability, and time constraints [49]. The Dietary Approaches to Stop Hypertension (DASH) diet is the cornerstone of dietary recommendations for patients with hypertension, yet racial and ethnic minoritized populations face barriers to adherence including food availability, cost, and cultural familiarity [77,78]. Minoritized populations are more likely to live in food deserts, which are known to be associated with higher systolic blood pressure [79,80]. Finally, Black and Hispanic patients are less likely to participate in physical activity during leisure time, independent of socioeconomic class [81].

When lifestyle interventions are insufficient, pharmacotherapy is used to control blood pressure. Despite worse blood pressure control overall, an analysis of the National Health and Nutrition Examination Survey (NHANES) showed that Black patients had similar awareness of their hypertension diagnosis and were prescribed antihypertensive medication at higher rates than White patients, while Asian and Hispanic patients had less awareness of their diagnosis and treatment [82]. A later NHANES study using data from 2013–2018 corroborated these findings, showing that Black patients were more likely to be prescribed more medications for hypertension but still achieved lower rates of blood pressure control [83]. Among patients with AF, The Aggressive Risk Factor Reduction Study for Atrial Fibrillation (ARREST-AF) demonstrated that patients following catheter ablation were significantly more likely to have arrhythmia-free survival in the cohort undergoing risk factor modification including control of hypertension [84]. A recent randomized clinical trial of this cohort showed that aggressive risk factor management, including achieving a target blood pressure <130/80 mm Hg at least 80 % of the time, reduced 1-year arrhythmia recurrence in patents post catheter ablation[85]. Nonetheless, to date there has been limited study of the role of hypertension management in reducing racial and ethnic disparities in adverse outcomes for patients with AF.

## 4 AF and diabetes mellitus

The prevalence of diabetes worldwide has risen from 200 in 1990 to 830 million individuals in 2022, with over 38 million people in the US carrying a diagnosis[86,87]. Racial and ethnic differences in diabetes prevalence exist, with rates ranging from 13.6 % for American Indian or Alaska Native (AI/AN), 12.1 % for Black, 11.7 % for Hispanic, 9.1 % for non-Hispanic Asian (Asian), to 6.9 % in White individuals[87,88]. Patients with diabetes have a 34 % higher risk of developing AF, compared to those without diabetes[89,90]. In a cohort of 293,124 individuals with type II diabetes in the VA, AF occurred in 14.9 % patients[91,92]. In the Framingham Heart Study, this number was reported to be 17.9 %, with the risk of developing AF observed as 3 % higher for each additional year of diabetes duration[90,93].

There are several mechanisms by which diabetes is thought to contribute to AF incidence (Fig. 2)[94,95]. Chronic hyperglycemia and insulin resistance promote atrial fibrosis, enlargement, and myocyte hypertrophy related to oxidative stress, advanced glycation end products and inflammation[92,94,[96], [97], [98], [99]]. Ultimately, these factors lead to cellular senescence and electrical remodeling which promote AF[100].

Diabetes is associated with poor outcomes for patients with AF including thromboembolic events and is associated with a 70 % increase in risk of stroke[101,102]. Prior studies have also demonstrated a dose-dependent risk of ischemic stroke and mortality with higher HbA1c levels, even after adjusting for stroke risk and anticoagulation. An analysis of Olmsted County, Minnesota residents with incident AF from 1980 to 2010 found that patients with diabetes had a higher risk of stroke, adjusted HR 1.32 (95 % CI 1.02–1.69), compared to those without diabetes[103]. Beyond stroke, diabetes confers a mortality risk in AF. Using data from the Outcomes Registry for Better Informed Treatment of Atrial Fibrillation (ORBIT-AF) registry, researchers found that patients with AF and diabetes had a significantly higher mortality risk, adjusted hazard ratio 1.63 (95 % CI of 1.04–2.56), compared to those without diabetes[104,104]. Diabetes was also associated with higher risk of all cause and cardiovascular hospitalizations in patients in this AF registry[89,104]. Beyond stroke and mortality, diabetes can also worsen symptom burden of AF and ultimately lead to heart failure as an additional comorbidity[105,106].

Control of diabetes is associated with improved outcomes in AF, including reduced incidence and recurrence. Prior research has shown that medications like metformin (Glucophage), thiazolidinediones, and dapagliflozin reduced the risk of AF and protected patients with diabetes from new-onset AF [96,[107], [108], [109]]. Sodium glucose cotransporter-2 inhibitors (SGLT2is) have also been shown to be associated with lower risk of incident AF compared to GLP-1RA therapies, though both were shown to reduce AF risk compared to placebo[110,111]. SLGT2 inhibitors have also been associated with decreased rates of heart failure related hospitalization or cardiovascular death in AF and, therefore, could be used as a cardioprotective medication in AF patients with diabetes[112]. Improved glycemic control prior to catheter ablation has also been shown to decrease risks of AF recurrence[31].

Despite evidence of the benefits of diabetes control in AF, the optimal stroke prevention strategies in patients with AF and diabetes has not been established[105]. Furthermore, pervasive disparities have long existed in diabetes care, especially within Black, Hispanic, and Asian American populations[113]. Hispanic and Black individuals are significantly less likely to achieve glycemic control compared to White individuals when diagnosed with diabetes[[114], [115], [116]]. Minoritized groups consistently have lower rates of SGLT2 inhibitors and GLP-1RA therapy receipt[117,118]. Among 1,197,914 U.S. Veteran patients with diabetes, Black patients had the lowest odds of prescription for GLP-1 RAs while Hispanic patients had the lowest odds of prescription for SGLT2is[119]. While there are multiple large cohort studies and meta-analyses examining the relationship between diabetes treatment and AF, there are no studies directly evaluating the role of variation in diabetes treatment by race, sex, socioeconomic status or other underrepresented identity on AF outcome disparities. Studies do, however, emphasize how minoritized populations are underrepresented in trials evaluating novel anti-diabetic therapies and their effect on cardiovascular outcomes[60,120]. Future work is needed to develop, test, and implement interventions to address the social and medical needs of patients with diabetes and AF to advance the care of this high-risk population[121].

## 5 AF and obstructive sleep apnea

The prevalence of obstructive sleep apnea (OSA) has increased significantly in recent years with an estimated 30 million U.S. adults and 1 billion worldwide with a diagnosis[122,123]. The percentage of White men and women diagnosed with OSA is 6.9 % and 3.2 %, respectively. In comparison, 4.4 % and 3.5 % of Black men and women, as well as 2.4 % and 1.4 % of Mexican-American men and women, are diagnosed with OSA in the U.S[124]. OSA in Black adults has been shown to increase the odds of AF by 58 %, compared to a 12 % increase in White adults[125].

The data surrounding OSA as a risk factor in the development of AF is abundant. In a study of 14 Canadian arrhythmia clinics, OSA was detected in 85 % of patients with AF[126]. Data from hospital records and follow-up phone interviews with patients with AF referred to Mayo Clinic Cardioversion Center shows that OSA increases the 12-month recurrence of AF after cardioversion from 53 % (patients without OSA) to 82 % (patients with untreated OSA)[127]. In addition to AF, OSA significantly increases the risk of stroke with, greater severity of sleep apnea at baseline associated with greater the risk of stroke. An observational cohort study of 1022 patients being evaluated for sleep-disordered breathing at the Yale Center for Sleep Medicine found that even after adjustment for various confounding variables, such as age, BMI, and hypertension, OSA was significantly associated with stroke[128]. Outcome data from a study of 53 patients with OSA showed that the majority of cardioembolic strokes occurred in moderate to severe cases of OSA. Within a year of the study, 72 % of subjects with OSA suffered a cardioembolic stroke, compared to 33 % of controls[129].

Nonetheless, the data on OSA and adverse AF outcomes is mixed. An analysis of patients with AF and OSA in Rome, Italy found that while OSA and AF were highly prevalent concurrently, OSA was not an independent risk factor for stroke in this patient population[130]. A broader European cohort of 364 patients with known AF and OSA found that indices of hypoxia during sleep were associated with a higher CHADS_2_VA_2_Sc stroke risk score, with the lowest level of hypoxia being associated with the highest level of cardiometabolic risk[131]. These studies suggest the importance of addressing OSA in patients with AF while representing a literature gap.

Despite the rising prevalence of OSA, the syndrome remains underdiagnosed in patients with AF. A prospective study of two tertiary U.S. hospitals included 188 patients with AF that did not have a prior diagnosis of sleep apnea[132]. Results from individual home sleep apnea tests showed that 82 % of these patients were actually positive for sleep apnea[133]. Once diagnosed, treatment of OSA in critical. A nurse-led program at the University of Pennsylvania demonstrated a potentially sustainable model for treating OSA, as patients showed improved quality of life and arrhythmia symptoms after 6 months[134]. Interventions such as these are significant because untreated OSA reduces the effectiveness of AF treatment including electric cardioversion, ablation, and pharmacological therapies[133,135].

Once OSA has been screened and diagnosed, the use of continuous positive airway pressure (CPAP) therapy is the preferred treatment as it has been associated with lower prevalence of arrhythmias and reduced sympathetic nerve activity[136]. In a prospective study of 720 patients from 2005–2011, AF recurrence was 35 % in patients with OSA using CPAP and 68 % in patients with untreated OSA[137]. In the ORBIT-AF study, AF progression in 1841 patients with OSA and AF was assessed. In CPAP users, AF progression was significantly lower in comparison to CPAP non-users[138]. Furthermore, in-lab studies have shown that there is a significant decrease in the atrial and ventricular ectopy count in patients with both OSA and AF after 3 and 6 months of CPAP treatment[139].

Notably, the data surrounding racial and ethnic differences in adherence to CPAP therapy is mixed. A multi-center clinical trial between five U.S. cities demonstrated lower CPAP acceptance rates in Black versus non-Black participants[140]. In contrast, researchers measured the mean number of self-reported hours per week of CPAP use among Black and White patients in order to assess CPAP acceptance rate, which showed similar rates between groups[141]. Access to care and health insurance are potential structural barriers in the treatment of OSA for minoritized groups. A recent review found that Black individuals are disproportionately affected by OSA due to underdiagnosis, lower rate of referral to sleep specialists, and lower adherence to CPAP therapy compared to white individuals[125]. This raises the possibility of OSA as a risk factor for AF complications that has higher implications for Black than White individuals. Nonetheless, there is a lack of research surrounding the racial and ethnic disparities that exist in treating OSA in patients with AF. Of particular concern, Black adults are markedly underrepresented in sleep related clinical studies[142,143]. This observation, an assessment of how biological, genomic, and social factors contribute to race-based differences in OSA and AF, and a clearer understanding of the role of medications, such as GLP1-RA therapies, in the management of OSA, overall, and for racial and ethnic minoritized populations, represent ample opportunities for future research.

## 6 AF and alcohol use

Alcohol consumption is a significant risk factor for AF (Fig. 2)[144]. Approximately 57 % of American adults drink regularly [145], though the prevalence of alcohol use in AF is notably lower. A study analyzing national trends in AF hospitalizations found that 4.29 % of AF hospitalizations were associated with alcohol use disorder (AUD)[146]. The 2023 ACC/AHA/ACCP/HRS Guideline for the Diagnosis and Management of Atrial Fibrillation highlights that even moderate alcohol consumption can elevate the risk of AF episodes, with an increase in risk observed within four hours of consuming a single drink [147], and heavier alcohol consumption predicting higher risk[[148], [149], [150]]. Alcohol can also lead to left atrial enlargement and fibrosis which both are known precipitators of AF risk[[151], [152], [153]]. Chronic heavy drinking further exacerbates this risk, contributing to the development and progression of AF[31]. As such, there is a Class 1B recommendation that patients with AF should decrease or eliminate alcohol consumption to limit recurrence and ectopy burden[31].

The association between alcohol consumption and adverse AF outcomes has been limited. An analysis using Korean nationwide claims data from 2010–2016 found that alcohol abstainers and non-drinkers had a lower risk of stroke than current drinkers[154]. Using data from the Global Burden of Disease Study 2021, researchers found that AF attributable to high alcohol use contributed to 11,908 deaths and 362,698 disability-adjusted life years across 204 countries worldwide[155]. These numbers notably represented 176.4 % and 132.9 % increases respectively since 1990, highlighting an urgent need to address this critical risk factor in AF care.

There are several pharmacotherapies that have been proven to be effective in alcohol cessation efforts including naltrexone, an opioid receptor antagonist, acamprosate, an NMDA receptor antagonist, disulfiram, a daily medication that inhibits acetaldehyde dehydrogenase, baclofen, gabapentin (Neurontin) and topiramate (Topamax tablets) which each act on the GABA pathway[156,157]. Recent evidence also shows that low-dose semaglutide reduced alcohol consumption, drinks per drinking day, weekly cravings, and heavy drinking over time in a randomized trial, highlighting its potential as a pharmacologic adjunct for alcohol use reduction[158].

Alcohol abstinence has been shown to significantly reduce the recurrence and burden of AF in regular drinkers with the condition. In a multicenter prospective randomized controlled trial, patients who abstained from alcohol had a lower AF burden and fewer recurrences compared to those who continued drinking (HR 0.55, 95 % CI, 0.36 to 0.84; *P* = .005) and over 6 months the AF burden in the abstinence group was less than half the control group (*P*=.01)[145]. There have been additional studies that also demonstrate lower AF burden in those who abstain including the Atherosclerosis Risk In Communities (ARIC) Study that demonstrated a 20 % lower incidence rate of AF for every decade abstinent from alcohol, a 13 % higher rate of AF for every additional decade of past alcohol consumption (and every additional drink per day during former drinking with a 4 % higher rate of AF[159]. Nonetheless, despite their benefit in alcohol abstinence, the direct effects of alcohol cessation medications on AF related outcomes have not been specifically studied, representing an opportunity for future study.

Racial disparities in alcohol consumption and its management may influence variation in AF associated outcomes. Studies have shown that minoritized groups, such as Black and Hispanic individuals, often face higher rates of alcohol-related health issues due to targeted marketing of alcohol in their communities and limited access to healthcare resources for managing AUD[[160], [161], [162]]. A study found that Black (aOR=0.78, 95 % CI=0.69–0.89) and Hispanic (aOR=0.75, 95 % CI=0.64–0.88) individuals were less likely to receive medication for AUD than their White counterparts[160]. Uninsured individuals and underinsured individuals were also less likely to receive less AUD medications as confirmed in additional studies[160,163].These disparities are compounded by socioeconomic factors, leading to a higher burden of alcohol-related cardiovascular conditions, including AF, in these populations[162,164,165]. However, no studies to date have specifically examined racial, ethnic, and socioeconomic disparities in alcohol cessation nor identified interventions to address such disparities in alcohol cessation in patients with AF.

## 7 AF and tobacco use

Smoking is a well-established risk factor for AF, with both current and former smokers demonstrating a higher risk of incident AF compared to non-smokers in a dose-dependent manner[93,166]. In the general population, the prevalence of cigarette smoking among U.S. adults was reported to be 11.5 % in 2021 [167], while the prevalence of smoking among individuals with AF is significantly higher[168]. According to a study from the Danish Diet, Cancer, and Health cohort, 34 % of patients with incident AF were current smokers, and 37 % were former smokers[168]. The ARIC study found that current smokers had a higher incidence of AF, with a population attributable fraction of 9.78 % for current smokers[93,169,170]. Another study utilizing the National Inpatient Sample database reported that 23.5 % of AF hospitalizations involved patients with tobacco use disorder[146].

Smoking affects the progression of AF through several mechanisms including promoting atrial fibrosis by upregulating transforming growth factor-beta1 (TGF-β1) and its receptor, and leading to increased collagen production, impairing atrial contractility (Fig. 2)[31,[171], [172], [173], [174]]. This structural remodeling creates a substrate conducive to AF. Smoking also increases oxidative stress and inflammatory signaling, which contribute to atrial structural abnormalities and remodeling[31]. Nicotine can also affect the autonomic nervous system, increasing sympathetic activity and reducing parasympathetic tone and exacerbating AF[175].

The literature suggests that smoking is associated with higher AF recurrence rates following catheter ablation, less time in therapeutic range on warfarin and therefore increased risk of stroke, heart failure, hospitalization and death[168,[176], [177], [178], [179], [180], [181], [182]]. A Danish cohort demonstrated that even among those receiving anticoagulation, the hazard ratios (HRs) (95 % CI) of thromboembolism or death were 3.13 (1.72–6.37) and 2.73 (2.02–3.70) among women and men who were currently heavy smokers respectively[183]. Another cohort of patients with AF in Japan found the adjusted HRs (95 % CIs) of current smokers relative to non–current smokers for ischemic stroke and all-cause mortality were 1.65 (1.03–2.64), 0.52 (0.12–2.15), and 1.26 (0.83–1.92), respectively. Despite the high rates of smoking in the U.S., an examination of the association of tobacco use on AF-related outcomes in this population is scarce.

There is currently a Class 1B recommendation for smoking cessation of patients with a history of AF who should receive cognitive behavioral therapy and a combination of pharmacologic interventions including varenicline, nicotine replacement therapy (NRT), and bupropion for tobacco cessation to mitigate adverse outcomes and cardiac complications[31]. Yet, smokers with AF are less likely to receive cessation intervention attempts than smokers without it[184,185]. Notably, GLP-1RAs, such as low dose semaglutide, are associated with reductions in cigarettes per day among current smokers, suggesting potential dual benefits for tobacco and alcohol use interventions[158].

Despite recommendation, myriad disparities exist in smoking prevalence and cessation efforts. Black patients have lower overall smoking rates compared to White patients but still experience a disproportionately high burden of smoking-related cardiovascular morbidity and mortality[186]. This disparity is partly due to certain social determinants of health, such as lower access to smoking cessation resources for Black, Hispanic, Asian, and uninsured smokers, targeted marketing of tobacco products, and higher levels of stress and socioeconomic disadvantage[[186], [187], [188], [189]]. In a study using the NCDR PINNACLE registry, significant provider-level variation in smoking cessation assistance (generally decreased) was observed in patients with AF compared to without, and these disparities were exacerbated based on location and patient demographics like race[184]. Another study conducted in primary care clinics found that non-White patients were less likely to receive counseling or medications even after adjusting for other socioeconomic factors compared to White patients[189]. Another study that examined pharmacotherapy efficacy of NRT in Black and White patients found that Black smokers had 49 % lower odds (95 % CI 0.39–0.66, *p*<.001) of achieving smoking cessation compared to their White counterparts, even after accounting for baseline smoking behavior, and adherence to treatment[190].

A Korean study found that male patients with newly diagnosed AF that were able to quit smoking had a 35 % reduced risk of CVD (aHR 0.65, 95 % CI 0.44–0.97) and a 41 % reduced risk of stroke (aHR 0.59, 95 % CI 0.35–0.99) compared to continual smokers[179]. In another study, patients from the Korean National Health Insurance Database with new-onset AF who quit smoking had lower risks of ischemic stroke and all-cause death compared to current smokers [191],. Among 97,637 patients, 6.9 % stopped after AF diagnosis and after 5-year follow-up this group had lower risk of ischemic stroke (HR 0.70, 95 % CI 0.59–0.83) and all cause death (HR 0.84, 95 % CI 0.75–0.95) than current smokers[191]. Nonetheless, while the 2023 ACC/AHA/ACCP/HRS Guideline for the Diagnosis and Management of Atrial Fibrillation stresses the importance of addressing health inequities and social barriers to AF management, including smoking cessation, there are no studies to date that examine the role of unequal receipt of smoking cessation therapies on AF associated outcome inequities[31].

## 8 Future research directions and call to action

Our review has highlighted myriad opportunities to extend the current literature and our understanding of the role of risk factors on adverse AF outcomes, the determinants of these disparities in these risk factors (Fig. 3) and how such risk factors are differentially managed among minoritized populations. To help advance this literature, racial and ethnic minoritized groups need greater representation in clinical trials and epidemiologic studies examining the medical and procedure-based management of AF[192]. Further, more research is needed on the effective and equitable implementation of lifestyle and risk factor modification in AF, particularly in individuals from racial and ethnically diverse backgrounds[30]. To date no such studies are present in the literature. In the management of obesity, research is needed to examine the role that GLP-1RA therapies and other weight loss therapies broadly may have on prevention of AF and its complications, particularly in traditionally underserved populations. In hypertension management, examining the role of newer agents including aldosterone synthase inhibitors and injectable angiotensinogen inhibitors which are on the horizon and how the diffusion of such therapies may influence AF-related stroke and mortality is needed[193,194]. To date there are no studies examining the role of diabetes control in minoritized patients and AF related complications, despite the availability of novel, effective therapies for manage diabetes, which represents a key target for future study. Evidently, early systematic screenings, proper diagnosis, and effective treatment of OSA, including with weight management, can greatly improve AF risk modification, and lead to improvement in the quality of life of patients, yet research in this field is scarce, highlighting another important area of investigation. Finally, addressing disparities in alcohol and tobacco cessation and their influence on AF-related complications requires not only research but targeted public health interventions and tailored strategies focused on culturally sensitive education and expanded access to care in nontraditional settings such as community centers or faith-based organizations[195,196].

To address the critical gaps in the literature described above, there is an urgent need for improved collection of social barriers to equitable risk factor management along with culturally directed, multilevel (e.g., patient, provider, health system, and policy) interventions to improve management of AF and stroke related risk factors (Fig. 4). Interventions to address systemic factors and structural and social determinants such as housing, education, employment, access to healthy food, insurance status, health, language, and digital literacy, perceived discrimination, transportation, environmental exposures, family and financial support are needed to help target non-healthcare factors that inform care access and quality[192]. Such interventions will only come through deep health system investments and shifting incentives to address social factors. Policies such as Medicaid expansion and the Inflation Reduction Act, which aim to reduce medication costs among low-income and older adults, can help shift funding to prioritizing these aforementioned social barriers[197]. Within health care, expanded training for clinicians to prioritize risk factor management in the care of AF while enhancing the workforce to ensure they are competent to address various backgrounds will improve care across each of these highlighted conditions[160,196]. Novel integrated care models, such as AF centers of excellence, that combine cardiovascular and risk factor treatments can provide comprehensive care for patients with AF and improve adherence to both AF and other risk factor treatments[198]. The involvement of a multidisciplinary team, including cardiologists, nurse practitioners, primary care providers, pharmacists, and social workers, can enhance the support for risk factor management provided to patients. Finally, an expanded focus on community partnerships can build trustworthiness and help health systems more effectively engage with groups that have traditionally had unequal access to health care.

## 9 Conclusions

In this review we highlighted several important mechanisms by which risk factors influence AF incidence and associated adverse outcomes. We reveal opportunities for further study within each risk factor to better understand how such factors are associated with disparities in AF outcomes. Regularly monitoring and evaluating the effectiveness of interventions and treatment outcomes across different racial and ethnic groups can help identify and address ongoing disparities. By implementing these strategies, healthcare systems can work towards eliminating racial and ethnic risk factor disparities among patients with AF, ultimately improving cardiovascular outcomes and overall health equity.

## Funding

This study is supported in part by a Robert Wood Johnson / American Heart Association Amos Medical Faculty Development Program Award.

## Disclosures

There are no disclosures to report.

**Fig. 1:**
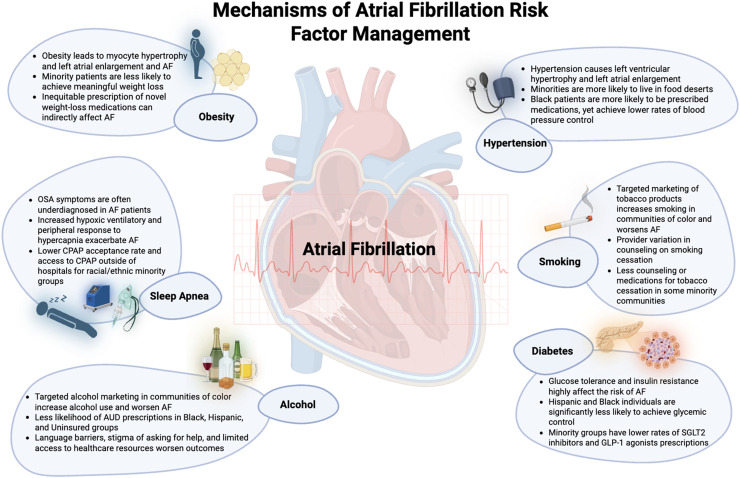
(Central Illustration). Mechanisms of Atrial Fibrillation Risk Factor Management.

Fig. 1 (Central Illustration). Mechanisms of Atrial Fibrillation Risk Factor Management

This figure represents how risk factors including obesity, sleep apnea, alcohol use, hypertension, smoking, and diabetes, have overlapping contributions to the pathophysiologic underpinnings of atrial fibrillation (AF). It further describes racial and ethnic differences in the management of these risk factors which in turn influences AF outcomes.

**Fig. 2:**
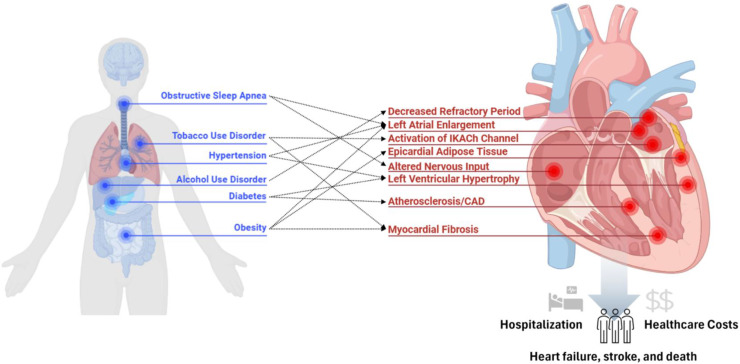
Cardiovascular Effect of Atrial Fibrillation Risk Factors.

Fig. 2. Cardiovascular Effect of Atrial Fibrillation Risk Factors

This figure highlights the key mechanisms contributing to disparities in the management of atrial fibrillation (AF), focusing on six major health factors: obesity, sleep apnea, alcohol use, hypertension, smoking, and diabetes. The diagram illustrates how these conditions exacerbate AF through distinct pathophysiological pathways at the cellular, organ, and individual levels. Disparities in the recognition and treatment of AF in populations with these risk factors are also emphasized, showing how socioeconomic, cultural, and healthcare system factors can exacerbate management challenges. The figure aims to provide a comprehensive overview of how each factor contributes to both the clinical outcomes and health inequities in AF patients.

**Fig. 3:**
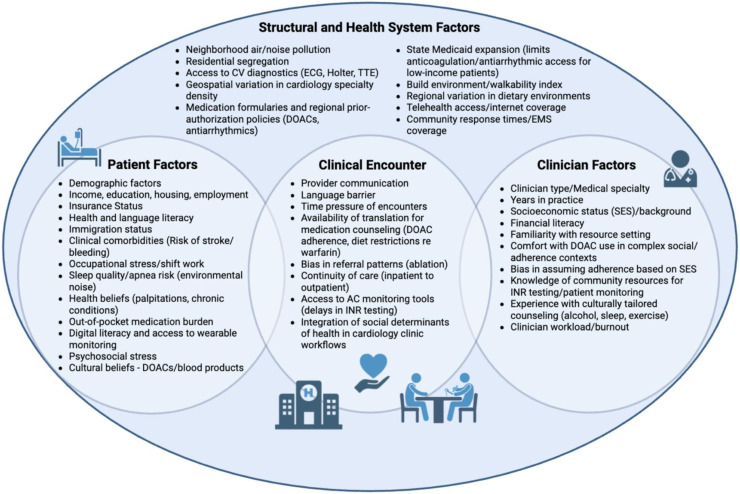
Determinants of Disparities in Atrial Fibrillation Risk Factors.

Fig. 3. Determinants of Disparities in Atrial Fibrillation Risk Factors

This figure represents a conceptual model for the multifaceted drivers of racial and ethnic disparities in risk factor management of patients with atrial fibrillation, including at the structural and health system, patient, clinician, and clinical encounter levels.

**Fig. 4:**
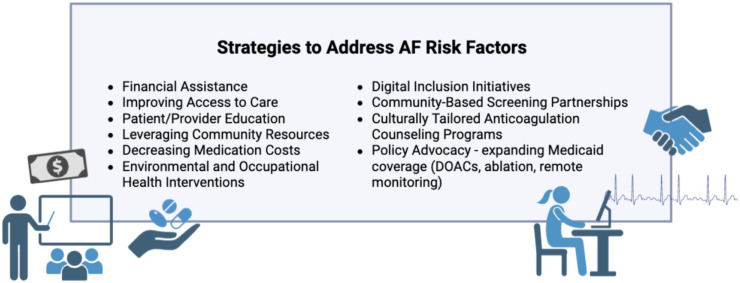
Atrial Fibrillation Risk Factors Targeted Solutions.

Fig. 4. Atrial Fibrillation Risk Factors Targeted Solutions

This figure provides strategies addressing disparities in AF risk factors through multifaceted solutions, including patient, policy, financial, and community level approaches.

## CRediT authorship contribution statement

**Anthony D’Oro:** Writing – review & editing, Writing – original draft, Visualization, Formal analysis, Conceptualization. **Zoee D’Costa:** Writing – review & editing, Writing – original draft, Visualization, Formal analysis. **Jasmyn J. Tang:** Writing – review & editing, Writing – original draft, Project administration, Formal analysis. **Stephen Min:** Writing – review & editing, Writing – original draft, Formal analysis. **Larry R. Jackson:** Writing – review & editing, Supervision, Formal analysis. **Utibe R. Essien:** Writing – review & editing, Writing – original draft, Visualization, Supervision, Funding acquisition, Formal analysis, Conceptualization.

## Declaration of competing interest

The authors declare that they have no known competing financial interests or personal relationships that could have appeared to influence the work reported in this paper.
